# ERIC recommendations for *TP53* mutation analysis in chronic lymphocytic leukemia—2024 update

**DOI:** 10.1038/s41375-024-02267-x

**Published:** 2024-05-16

**Authors:** Jitka Malcikova, Sarka Pavlova, Panagiotis Baliakas, Thomas Chatzikonstantinou, Eugen Tausch, Mark Catherwood, Davide Rossi, Thierry Soussi, Boris Tichy, Arnon P. Kater, Carsten U. Niemann, Frederic Davi, Gianluca Gaidano, Stephan Stilgenbauer, Richard Rosenquist, Kostas Stamatopoulos, Paolo Ghia, Sarka Pospisilova

**Affiliations:** 1 Department of Internal Medicine, Hematology and Oncology, and Institute of Medical Genetics and Genomics, University Hospital Brno and Medical Faculty, Masaryk University, Brno, Czech Republic; 2 Central European Institute of Technology, Masaryk University, Brno, Czech Republic; 3 Department of Immunology, Genetics and Pathology, Uppsala University, Uppsala, Sweden; 4 Institute of Applied Biosciences, Centre for Research and Technology Hellas, Thessaloniki, Greece; 5 Division of CLL, Department of Internal Medicine III, Ulm University, Ulm, Germany; 6 Haematology Department, Belfast Health and Social Care Trust, Belfast, United Kingdom; 7 Hematology, Oncology Institute of Southern Switzerland and Institute of Oncology Research, Università della Svizzera Italiana, Bellinzona, Switzerland; 8 Hematopoietic and Leukemic Development, UMRS_938, Sorbonne University, Paris, France; 9 Department of Hematology, Cancer Center Amsterdam, Amsterdam University Medical Centers, Amsterdam, the Netherlands; 10 Department of Hematology, Rigshospitalet, Copenhagen, Denmark; 11 Sorbonne Université, Paris, France; 12 Department of Hematology, Hôpital Pitié-Salpêtière, AP-HP, Paris, France; 13 Division of Haematology, Department of Translational Medicine, University of Eastern Piedmont, Novara, Italy; 14 Department of Molecular Medicine and Surgery, Karolinska Institutet, Stockholm, Sweden; 15 Clinical Genetics and Genomics, Karolinska University Hospital, Stockholm, Sweden; 16 Università Vita-Salute San Raffaele, Milan, Italy; 17 Strategic Research Program on CLL, Division of Experimental Oncology, IRCCS Ospedale San Raffaele, Milan, Italy

**Keywords:** Risk factors, Oncogenes

## Abstract

In chronic lymphocytic leukemia (CLL), analysis of *TP53* aberrations (deletion and/or mutation) is a crucial part of treatment decision-making algorithms. Technological and treatment advances have resulted in the need for an update of the last recommendations for *TP53* analysis in CLL, published by ERIC, the European Research Initiative on CLL, in 2018. Based on the current knowledge of the relevance of low-burden *TP53*-mutated clones, a specific variant allele frequency (VAF) cut-off for reporting *TP53* mutations is no longer recommended, but instead, the need for thorough method validation by the reporting laboratory is emphasized. The result of *TP53* analyses should always be interpreted within the context of available laboratory and clinical information, treatment indication, and therapeutic options. Methodological aspects of introducing next-generation sequencing (NGS) in routine practice are discussed with a focus on reliable detection of low-burden clones. Furthermore, potential interpretation challenges are presented, and a simplified algorithm for the classification of *TP53* variants in CLL is provided, representing a consensus based on previously published guidelines. Finally, the reporting requirements are highlighted, including a template for clinical reports of *TP53* aberrations. These recommendations are intended to assist diagnosticians in the correct assessment of *TP53* mutation status, but also physicians in the appropriate understanding of the lab reports, thus decreasing the risk of misinterpretation and incorrect management of patients in routine practice whilst also leading to improved stratification of patients with CLL in clinical trials.

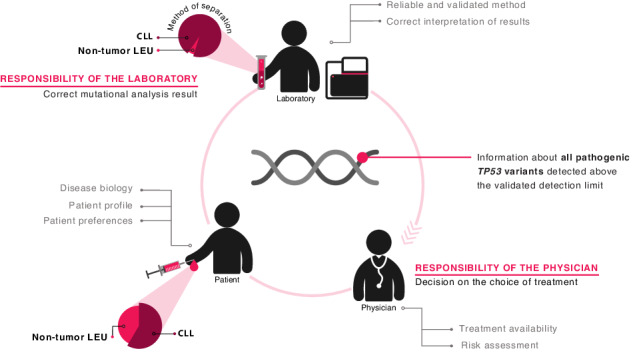

## Clinical impact of *TP53* alterations in patients with CLL

**Fig. 1:**
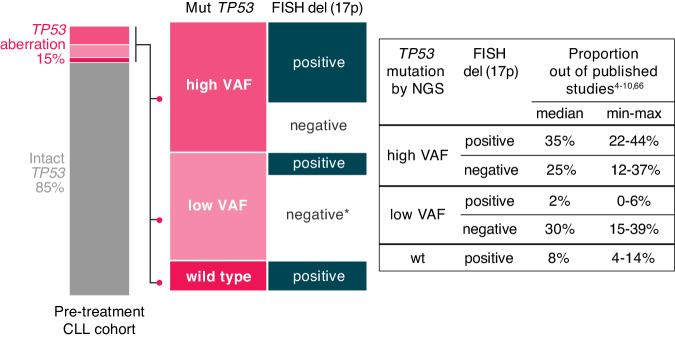
Breakdown of *TP53* aberrations detected in CLL based on the presence of *TP53* mutations, their allele burden, and concomitant del(17p) as assessed by FISH. Values were adopted from published studies employing ultra-deep NGS to detect *TP53* mutations [4–10, 66]. High VAF—variants >10% VAF, low VAF—variants 1–10% VAF, except for two studies where variants <1% and >1% could not be distinguished [4, 5]. In patients with high VAF *TP53* mutations, co-existence of del(17p) prevails. * In patients carrying low VAF *TP53* mutation concomitant del(17p) is detected in only a minority of cases, but the true status is unknown due to the higher detection limit of FISH (>5% aberrant nuclei). The breakdown depicted here corresponds to pre-treatment cohorts (diagnosis or before frontline treatment). In the chemo-pretreated cohorts the proportion of patients with *TP53* defects can reach 40% [1, 20, 106].

*A TP53* aberration is defined as either the deletion of the *TP53* gene locus on 17p13 [del(17p)] or the presence of a mutation, i.e., somatic change in the sequence of the *TP53* gene (*TP53*mut). The frequency of *TP53* aberrations in patients with chronic lymphocytic leukemia (CLL) is higher in those with unmutated immunoglobulin heavy variable (IGHV) genes. Generally, the frequency is low at diagnosis (5-10% of patients, depending on the method used), it is slightly higher in cohorts of patients entering frontline treatment (10–20%; Fig. 1), and further increases in later disease stages, predominantly in chemoimmunotherapy (CIT)-treated patients and Richter transformation (up to 50%) [1–3]. In patients with CLL, del(17p) is mostly accompanied by *TP53* mutations, and sole del(17p) is infrequent, while sole *TP53* mutations are more commonly found (Fig. 1) [4–10].

### Prognostic value of *TP53* alterations

In the early 1990s, several studies reported the prognostic relevance of *TP53* aberrations [11–14]. Subsequently, in the Döhner hierarchical model, del(17p) was classified as the most adverse cytogenetic abnormality [15]. These findings were further underpinned by many studies [16–19], including clinical trials [20–22], highlighting the independent role of both del(17p) and *TP53* mutations.

The prognostic value of *TP53* aberrations is evident early in the course of CLL. Several prognostic scores developed to predict time-to-first-treatment (TTFT) include *TP53* aberrations as a variable. In the CLL1 trial, del(17p) conferred a shorter TTFT and was given the highest score in a weighted point system of variables (CLL1 prognostic model) [23, 24]. Similarly, the CLL international prognostic index (CLL-IPI) and the CLL WithOut Need of Treatment (CLL-WONT) incorporate *TP53* aberrations as an independent predictor of shorter TTFT [25, 26]. Conversely, *TP53* aberrations failed to predict TTFT in the training cohort of the International Prognostic Score for Early-stage CLL (IPS-E) [27]. This finding was attributed to the differential impact of *TP53* aberrations on TTFT based on the mutational status of the IGHV genes. Further supporting this reasoning, a recent ERIC study and a single center study from MD Anderson revealed that *TP53* aberrations predict TTFT only in patients with unmutated IGHV genes [28, 29].

*TP53* aberrations also have paramount prognostic value in treated patients with CLL since, generally, they confer a worse prognosis with all available treatments, including agents targeting B cell receptor (BcR) signaling and BCL2, at least in the relapsed/refractory setting [30–33]. Interestingly, *TP53* aberration status may potentially affect targeted treatment outcomes differently compared to CIT. In particular, the prognostic value of single-hit *TP53* (isolated del(17p) or sole *TP53* mutation) remains unclear with targeted agents, while concomitant *TP53* mutations and del(17p) (multi-hit *TP53*) appear to be independently associated with worse outcomes in some of the studies [34–37]. However, since in many published studies del(17p) and *TP53* mutations were not distinguished [32, 33], and in some only del(17p) was included [38], this relevant issue is currently inconclusive. Moreover, the presence of homozygous mutations has not been considered at all. Thus, it is now imperative to include definitions of the type, clonal burden, and number of *TP53* defects in clinical trials and academic studies in order to be able to provide a uniform classification, similar to myeloid neoplasms [39].

### Predictive value of *TP53* alterations

The predictive value of *TP53* aberrations is clear when CIT regimens are included among the treatment options: in fact, targeted agents as either monotherapy or in combination outperformed CIT regimens in the frontline and R/R settings [33, 40–43] and represent the preferred option for these patients [44].

On the contrary, the role of *TP53* aberrations in choosing between targeted agent regimens is less well studied. In the ALPINE trial, zanubrutinib conferred a better PFS than ibrutinib in all R/R patients including those with del(17p)/*TP53*mut [45], while in the ELEVATE-RR trial, no superiority of acalabrutinib vs. ibrutinib was observed [46]. Except for these findings, conclusions about the predictive value of *TP53* aberrations are based on cross-trial comparisons in which the prognostic impact of *TP53* on PFS appears to be stronger with time-limited regimens [42, 47] than with continuous therapy [40]. Nevertheless, the lack of direct comparisons precludes definitive conclusions from being drawn at present.

### Relevance of low-burden *TP53* mutations

**Fig. 2:**
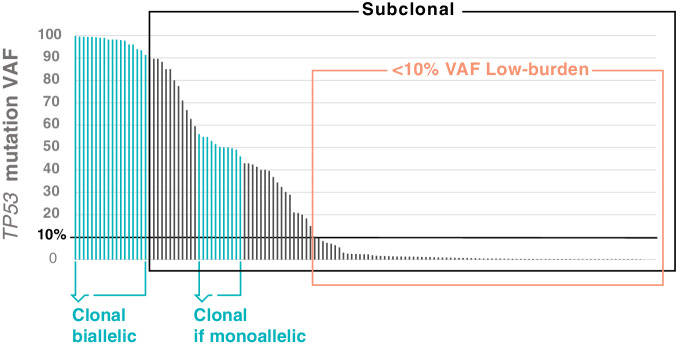
Illustrative example of clonal distribution of *TP53* variants. The distribution of variant allele frequencies (%VAF; *y*-axis) of *TP53* mutations detected in patients with CLL (*x*-axis). Variants present in the whole cancer population are clonal, otherwise, they are deemed subclonal. Variants <10% VAF are considered low burden. This distribution is valid when the sample contains >90% tumor cells. In samples with a low CLL cell fraction, a low VAF may, in reality, correspond to a clonal mutation.

The advent of next-generation sequencing (NGS) in routine practice allowed the detection of clones carrying variants below the detection limit of Sanger sequencing, which was arbitrarily set to 10% variant allele frequency (VAF). When referring to such clones (<10% VAF), it is recommended to use the terms “low-burden,” minor-clone,” “low-VAF,” or “low-level,” and to avoid the terminology “subclonal,” as this is generally used to describe variants not present in the entire tumor population, as opposed to “clonal” [48] (Fig. 2). Indeed, it is impossible to define the clonality of a *TP53* variant if the tumor fraction in the assayed tissue and the ploidy of the *TP53* locus are unknown, as is usually the case in molecular diagnostic laboratories.

The clinical relevance of low-burden *TP53* mutations is still debated. The vast majority of evidence was obtained in the era of CIT, and no clinical trial was designed to assess their impact. The conclusions are based mainly on retrospective studies comparing PFS and OS in patients with low-burden *TP53* mutations [4–7, 9] and in a single prospective clinical trial, albeit with a different initial endpoint [8]. The existing evidence mostly, but not uniformly, suggested shortened survival for patients with low-burden *TP53* mutations, with the median OS being intermediate between patients having high-burden *TP53* mutations and those with intact *TP53* [4–7, 9] (Supplementary Table S1). Differing prevailing types of treatment and cohort constitutions mainly contribute to the differences between studies. Some studies analyzed diagnostic or early-stage cohorts with higher proportions of patients with mutated IGHV genes, while *TP53* testing is generally indicated in active disease, where unmutated IGHV genes prevails. Prospective assessment of low-burden *TP53* mutations in CIT-treated patients is not expected as this type of treatment has been superseded by chemo-free approaches. Nevertheless, independent studies have consistently shown that the small *TP53-*mutated clones are at a high risk of clonal expansion when treated with genotoxic agents as in CIT regimens [5–7, 49, 50]. In contrast, targeted agents act independently of the p53 pathway and, as such, are assumed not to directly accelerate the expansion of *TP53* deficient clones. In line with that, no preferential pattern of clonal evolution of *TP53*-aberrant clones was described upon treatment with targeted agents, with all scenarios of clonal development being observed (persistence, expansion, and disappearance) [6, 50–55]. Nevertheless, the follow-up is short in many studies, and it is unclear how the *TP53*-aberrant clone will evolve after several lines of targeted agents and if the *TP53* defect can promote resistance via facilitating genomic instability. Thus, the clinical impact of low-burden *TP53* mutations in patients treated with targeted agents is yet to be defined [56].

From a technical standpoint, it is important to emphasize that not all low-VAF variants are truly low-burden, in particular when samples with a lower proportion of tumor cells are analyzed [57]. This applies especially to patients with small lymphocytic lymphoma (SLL) or patients with predominantly nodal relapse with limited lymphocytosis. For example, the variant detected in 10% VAF in the unpurified bulk sample can be fully clonal (i.e., present in all cancer cells) if the cancer cell fraction is 20% and there is no loss of heterozygosity [57].

**Fig. 3:**
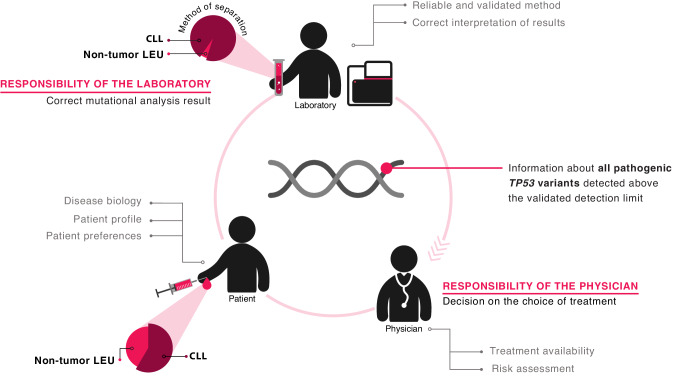
Responsibilities and cooperation between the laboratory and the physician with respect to *TP53* mutation diagnostics and interpretation. The laboratory is responsible for issuing the correct result and reports all pathogenic *TP53* variants above the validated LoD. The result should be interpreted in the context of tumor cell content, separation method, and disease status. The physician decides about the treatment based on all available information: the laboratory results, the clinical characteristics of the patient, patient preferences, and the availability of the treatment.

Altogether, the current consensus is that CIT should be strictly avoided in all patients with *TP53* aberrations, irrespective of the clone size. On these grounds, ERIC proposes that no limitation should be set for reporting regarding *TP53*-mutant clone size, while at the same time placing a strong emphasis on thorough methodological validation/ verification (Fig. 3). More particularly, laboratories should assess their own technical limit of detection and method performance, and describe them in the report (see section – “NGS-based approaches for *TP53* mutational analysis in CLL”). The result should always be interpreted in the context of tumor cell content, separation method, and disease phase. In this way, the *TP53* report will complement clinical information and patient preferences for an optimal treatment recommendation.

## Procedure description

### Methodology for *TP53* status evaluation

Fluorescence in-situ hybridization (FISH) should be employed for the detection of del(17p). A cut-off for a positive result (% of positive nuclei) needs to be assessed for each laboratory, sample type, and processing, and no generally applicable cut-off (e.g. 7%) can be given. Poor technical performance (e.g. low hybridization efficiency) may result in false-positive del(17p) calling. The procedure should follow the European Recommendations and Quality Assurance for Cytogenomic Analysis of Haematological Neoplasms [58]. The evaluation of del(17p) as a part of NGS-based strategy or array-based techniques is not recommended since the limit of detection for copy-number alterations (CNAs) is currently insufficient (~20% aberrant cells) and may lead to overlooking deletions present in lower cell fractions. It may, however, bring information on concurrent CNAs and disclose copy-neutral loss of heterozygosity (CN-LOH) of the *TP53* locus.

ESMO [59] recommends assessing del(17p) first and then *TP53* testing only in cases without del(17p). Following this two-step procedure can be difficult and may cause treatment delays but it may be reasonable in the presence of financial constraints. In addition, the knowledge about both abnormalities might be informative given the above-discussed issue of single vs. multi-hit *TP53* aberrations [35, 36, 60]. Therefore, it is preferred to analyze both *TP53* gene mutations and locus deletions simultaneously, if possible.

For *TP53* variant detection, the preferred methodology is NGS, but Sanger sequencing can still be used if NGS is not available. The main limitations of Sanger sequencing concern its low-throughput performance and the detection limit, that varies between 10–20% VAF and is dependent on sequence context, user experience, and software for the analysis of sequencing chromatograms [61]. Attention must be paid to checking the primers for the presence of population variants that lead to allelic drop-out and possible failure to detect the mutation (this applies to both Sanger sequencing and amplicon-based NGS). The list of population variants is expanding with increasing knowledge [62]. Such variants are present within the sequence of some of the previously recommended IARC protocol primers [63], and these should be used with caution (primers alongside with the information about the population variants are listed in Supplementary Table S2).

The basic approach valid for both Sanger sequencing and NGS for sampling, DNA isolation, and the covered region was described in the recommendations issued by ERIC in 2018 [64] and is still applicable. The basic principles are summarized in Table 1 including updates discussed below. The following text pinpoints the most important issues and reflects the recent developments in the sequencing methodology and resulting requirements for the quality of the testing, the interpretation, and the reporting.

**Table 1:** Overview of ERIC recommendations for *TP53* analysis.

ERIC recommendation				Notes and alternatives
Patients		Sampling	Always when deciding about treatment in both the frontline and the relapsed/refractory setting.	
Material		Type of material	Peripheral blood (PB)	Bone marrow, lymph nodes – suitable alternatives if PB lymphocyte count is low, e.g. in SLL/CLL, relapse in lymph nodes. Fresh/frozen tissues are strongly preferred.
		Tumor cell enrichment	Optimally separate CD19^+^ cells. Alternatively, choose the method of separation based on content of CLL cells, if the information about the blood count is available.	Separation of mononuclear cells is sufficient for most cases at treatment initiation. The separation of CD19^+^ lymphocytes is necessary when the proportion of CLL cells in the sample is low (ALC ≤ 10 × 10^9^/l)
		Nucleic acid	DNA	RNA analysis carries a risk of omitting truncating variants.
		Covered region	Optimum: exons 2-11 (coding region), Minimum: exons 4–10, Always include splice sites (at least ±2 intronic bp)	
Procedure	Sanger sequencing	PCR protocol	Check primer sequences for presence of population variants.	
		Sequencing	Both strands (forward + reverse)	
		Data analysis	Use software designed for somatic variant detection	Free web-based software GLASS [61] is accessible via ERIC website.
	NGS – preferred methodology	Library preparation	Amplicon or capture-based approaches are applicable. DNA input should be sufficient to achieve the aimed limit of detection.	Several ready-to-use kits involving *TP53* analysis are commercially available.
		Limit of detection (LoD)	Should be set to detect low-VAF variants ( ≤ 5% VAF).	Either variant-specific LoD or general LoD ensuring calling of >99% of all variants.
		Sequencing depth	Covering all bases in the coding region with a sufficient number of reads should be a standard.	≥99% minimum coverage percentage should be reported.
		Data analysis	Pipeline set to reliably distinguish variants from background noise	Commercial or in-house bioinformatics pipelines are applicable.
		Validation	Validate/verify the method before introducing it into diagnostics	Continuous monitoring of quality and external quality assessment is necessary.
Interpretation and reporting		Variant description	Use HGVS nomenclature: http://varnomen.hgvs.org/ [83] Report the cDNA and protein level including reference sequence.	
		Interpretation	Check the variant functionality in locus-specific databases: The TP53 database: https://tp53.isb-cgc.org/ [98] TP53 website: http://p53.fr/ [99] with embedded tool Seshat [85]	Interpretation algorithm provided as a part of these recommendations
		Populational and benign variants	It is preferred not to include (likely) benign variants in the report.	Check the variants with preserved functionality using gnomAD [62] and The ClinGen Evidence Repository of curated variants [88].
		VAF cut-off for reporting	Report all variants above the validated limit of detection.	The laboratory is responsible for issuing the correct result, clinical decision-making is within the responsibilities of the referring clinician
		Report form	Should follow ISO 15189 Medical laboratories — Requirements for quality and competence [68].	A template report form is available on the ERIC website.

*PB* peripheral blood, *BM* bone marrow, *ALC* absolute lymphocyte count, *NGS* Next-generation sequencing, *VAF* Variant allele fraction

### Sampling and enrichment of cancer cells

Tumor cells should be enriched to avoid VAF underestimation, or even missing a variant. Moreover, when non-separated leukocytes are analyzed using NGS with low detection limit, the detection of small *TP53*-aberrant clones not related to CLL, i.e., detection of clonal hematopoiesis of indeterminate potential (CHIP) [65], cannot be entirely excluded.

Based on the local practice, two approaches for cancer cell enrichment can be adopted. The optimal strategy is the separation of CD19^+^ cells in all CLL samples that can be performed via positive or negative selection. Negative selection is a more cost-effective approach in most CLL cases, yet might not be affordable for all laboratories. Alternatively, the referring physician provides the information about blood count (ideally, flow cytometry result) alongside the diagnosis and reason for referral, and the laboratory chooses the sample processing method based on tumor cell proportion and the limit of detection of the sequencing method. In that case, separation of mononuclear cells is satisfactory for most of cases at treatment initiation when the absolute lymphocyte count is usually high, while separation of CD19^+^ lymphocytes is performed only when the proportion of CLL cells in the sample is low (usually when ALC ≤ 10 × 10^9^/l, depending on the detection limit of the sequencing method and the aimed cut-off). If NGS with a low detection limit is used to detect variants in a sample with a low cancer fraction that has not been subjected to CD19^+^ cell enrichment, the VAF should be adjusted to the proportion of tumor cells.

We acknowledge that neither approach might be applicable in routine practice. When the laboratory does not receive the information on CLL cell content and routine CD19^+^ cell separation is not doable due to cost/time expenses, the laboratory should employ separation of mononuclear cells and inform the clinician in the report that the result should be interpreted with respect to tumor cells content in the provided sample.

In some circumstances, a lymph node or a bone marrow sample may also be used. In these cases, the content of tumor cells (typically in the pathology report) should be communicated between the clinic and the laboratory, and the knowledge is essential for the result interpretation.

### NGS-based approaches for *TP53* mutational analysis in CLL

Various commercial ready-to-use, custom, or entirely laboratory-developed approaches are used by different laboratories [66]. No specific methodology is recommended, and the laboratory is free to decide about the method based on resources and infrastructure (including computational resources), the focus of the laboratory (parallel analysis of other genes and diseases, minimal VAF to be detected), and legal requirements and reimbursement in the region [67]. In compliance with ISO 15189 standards for medical laboratories [68], all methods must be properly validated or verified (for details, see below). The EU-IVDR regulation [(EU) 2017/746] may increase the need for the use of commercial tests compliant with IVDR and the need for standardization of laboratory-developed tests.

The introduction of NGS methodology in the diagnostic routine is a complex process (Table 2); aspects to be considered are detailed e.g., in A Joint Consensus Recommendation of the Association for Molecular Pathology (AMP) and College of American Pathologists (CAP) [69] and in the guidelines issued by the Clinical and Laboratory Standards Institute (CLSI) [70]. Here, we summarize aspects that we consider worth highlighting specifically in the context of *TP53* mutation analysis in CLL.

**Table 2:** Implementation of NGS test in the diagnostics.

		Commercial IVD/IVDR/FDA assay^a^		Laboratory-developed test (in-house/custom)		
Process	Tools, material	Steps to be fulfilled		Steps to be fulfilled		Specifications and *TP53*-CLL-specific notes
Test familiarization	Explore the clinical and analytical needs	Define turnaround time, costs, number of samples, instrumentation, personnel, etc.				Standalone or panel assay
Test development	Study available technologies	Choose a commercial method		Test design		Regions to be sequenced Library preparation method Sequencing technology Bioinformatics
				Determine required test performance metrics and sequencing settings		Aim to detect low-VAF variants Consider: DNA input, coverage (each base of *TP53* gene has to meet the minimum read depth)
				Prepare SOP		
Optimization	Perform pilot run(s) Reference material Non-DNA controls	General assessment of method applicability for the purpose		Identify and solve errors and weaknesses		
				Check if the required parameters are met		
				Set the critical values (pass/fail criteria)		Output library quality and quantity Sequencing performance (level of background noise/overall error rate, coverage non-uniformity, low/high coverage, base call quality metrics etc.)
Verification/validation	Reference material (different from the material used during optimization)	**Verification**	Prepare verification protocol	**Validation**	Prepare validation protocol	
			Test the ranges provided by the manufacturer and technical variables in the laboratory		Include variables influencing assay performance	Amount of input DNA Number, type of samples in sequencing run Sequencing machine and other instruments Personnel
			Verify the values provided by the manufacturer		Assess parameters describing the test performance	Limit of blank, limit of detection Repeatability, reproducibility Proportion of true/false positives/negatives, etc. (See Supplementary Table 3)
Quality control (QC) and continuous monitoring	Diagnostic samples Regularly analyzed positive and negative controls	Monitor of specimen parameters				Sample purity, DNA quality, etc.
		Check variant profile				Proportion of *TP53*-mut/del(17p) and low-burden *TP53* mutations, variant profile (Figs. 1, 4, 6)
	Samples supplied by EQA provider	External quality assessment (EQA)				
	Samples provided by independent laboratory	Interlaboratory comparison				

^a^When using commercial research-use-only tests, the steps of test development and optimization are the same as for IVD/IVDR assays, but full validation must be performed.

#### Library preparation and sequencing strategies

Targeted NGS can be used to analyze the *TP53* gene as a standalone assay or as part of a gene panel investigating multiple genes. The method for detecting *TP53* variants in CLL should be designed to detect low-VAF variants. We recommend to aim at least at 5% VAF; methods can be optimized to a 1% VAF or even <1% VAF. However, it is currently technically challenging to distinguish true variants from background noise at such a limit of detection [57, 66, 71]. To reliably detect low-VAF variants, sufficient DNA input must be used. The sample must contain an adequate number of variant molecules that should be distinguished from background noise. No strict recommendation regarding input DNA can be given. The laboratory should consider the aimed detection limit, number of required variant reads and the library conversion rate i.e. the percentage of input alleles that is present in the sample after library preparation that can be sequenced, which differs significantly among the library preparation methods (10–70%). As an example, if the laboratory aims at 20 supporting reads and a detection limit of 1% VAF, the minimum number of alleles to be sequenced is 2000. Providing that the library conversion rate is 40%, the number of input alleles should be at least 5000, i.e. 2500 cells, corresponding to 15 ng of DNA (a diploid genome of a human cell corresponds approximately to 6 pg of DNA). As there is variance in each step (dilution, pipetting, amplification, sequencing), we would recommend at least twice as high DNA input, i.e. 30 ng in this particular example.

For library preparation, both amplicon- and capture-based methods can be used, each having pros and cons. Amplicon methods can detect low-VAF variants efficiently but might be problematic regarding the quantification of variants and allele drop-out. When using hybrid capture NGS, the risk of allele drop-out is minimized, albeit library conversion rate may be less efficient. Single primer extension (SPE) has a good library conversion rate and represents an effective approach used by several companies. Capture methods and SPE are also easily extendable to other targets. For more accurate quantification and PCR and sequencing error correction, using unique molecular identifiers (UMI) is useful [72].

The sequencing technology is a quickly evolving field, and the currently used technologies employ different approaches, generating different error profiles. Further development in this field is expected to decrease the error rate for both short-read and long-read sequencing in the near future.

For reliable calling of low-VAF variants, sufficient sequencing coverage must be achieved. The desired coverage depth should be determined based on the intended limit of detection and the error rate of the whole assay (sample processing, library preparation, and sequencing). According to the binomial data distribution, a coverage depth of 250 unique reads for each position should be sufficient to detect 5% VAF with a threshold of variant supporting reads ≥5 [69]. We consider this as an absolute minimum for each position, and laboratories are encouraged to aim at higher coverage (>750), since 5 reads supporting the variant is mostly insufficient, and the minimum required number of variant reads varies among different methods. It is imperative to monitor the minimal coverage for each position within the *TP53* coding region in each sequencing run. Importantly, this also pertains to the *TP53* gene sequenced as a part of a gene panel. Median or mean coverage is not informative as some positions could be sequenced with lower-than-required coverage, thus contributing to the possibility of false-negative and false-positive results. The median coverage should usually be at least twice as high as the target minimal coverage, but this highly depends on the coverage uniformity. Laboratories might use an online calculator to help set the coverage [71], but the parameters should be verified in subsequent steps. Importantly, employing UMI for consensus variant calling requires significantly higher coverage as the number of reads is reduced during the analytical process.

Additionally, the laboratory may employ other methods to reliably call low-VAF variants, such as dilution-based approach [9], repeating the analysis, and error suppression bioinformatics [73, 74].

#### Data analysis

The bioinformatics pipeline for NGS data analysis contains several steps, each of which can significantly influence the obtained results. Multiple commercial tools are available, some connected with the particular laboratory solution. Commercial tools are usually set to the safe, i.e., higher detection limit towards decreasing the risk of false positivity. Some of these tools allow changing the level of stringency; such change enables calling previously undetected variants but should be set with caution, and validated to prevent false-positive results. In-house bioinformatics pipelines are built based on multiple tools and can be adapted to individual needs, but they require an experienced bioinformatics team closely collaborating with the laboratory. Details of building and validation of in-house pipelines are out of the scope of this paper and can be found elsewhere [75–77].

The pipeline should provide an initial quality control summary including the coverage and other parameters, as it helps identify the samples with suboptimal results. The data generated by the bioinformatics pipeline should be carefully scrutinized focusing on technical artifacts that occur repeatedly within and among individual sequencing runs.

#### Validation/verification process

It is only acceptable to report laboratory results in clinical diagnostics after the method has been thoroughly validated or verified to ensure that the assay is suitable for its intended use, i.e., reliable detection of *TP53* variants [68, 69]. Commercially available CE-IVD/IVDR marked assays must be verified to confirm the manufacturer’s assay specifications using positive and negative controls with particular attention to the lowest VAF declared to be detected. Validation is a more detailed, multi-step process used for laboratory-developed, custom, and research-use-only (RUO) test, or CE-IVD assays used outside their designated range of use.

Certified reference material for thorough validation of somatic *TP53* variants, especially if those of <10% VAF are considered, is, unfortunately, unavailable. As reference material, the following can be used: (i) DNA from young, healthy controls; (ii) DNA from cell lines carrying known *TP53* variants (listed in the *TP53* database (https://TP53.isb-cgc.org/explore_cl), which could be diluted to various VAFs; (iii) tumor DNA from patients analyzed with an orthogonal method.

The validation phase should be preceded by the optimization step, which involves performing a pilot run(s) with well-characterized reference samples. During this step, unanticipated problems with an NGS test are identified, and critical values are set that trigger close evaluation and warn about the unreliability of the result (Table 2).

The validation process of the NGS method must be documented and should consider all possible variables that may influence the performance of the assay (Table 2). In the context of validation, parameters describing the test performance should be assessed (Supplementary Table S3). The terminology referring to the performance parameters was adopted from analytical chemistry and its transfer to NGS field resulted in inconsistency and confusion. Different meanings of the same term can be noted among clinical laboratories and also in various guidelines. This applies, in particular, for “limit of detection (LoD)”, “detection limit”, “sensitivity”, and “analytical sensitivity” that are sometimes used interchangeably, but are also used in several other ways (see the Clinical and Laboratory Standard Institute Harmonized Terminology Database: https://clsi.org/standards-development/harmonized-terminology-database/). Therefore, it is always recommended to include a brief explanation of the used term in the report. Here, we adopted the terminology and definitions according the Clinical and Laboratory Standard Institute [70].

As a first step, the background of the method must be assessed based on sequencing of DNA from young healthy controls. Based on the background distribution, the value that enables distinguishing true variants from background is set, usually referred as to Limit of Blank (LoB). Background noise is variant- and method-specific and consists of errors that may arise in each step of the sequencing process, i.e. library preparation, sequencing and bioinformatics processing. Also, background may be influenced by multiplexing of libraries of variable complexity due to index mis-assignment (index swap). It is generally low in non-patterned bridge-amplification platforms but still may affect ultra-sensitive approaches [78, 79]. Effect of index swap can be minimized by using unique dual indexing (UDI).

As a next step, the minimum allele fraction that can be confidently detected should be evaluated using serially diluted variant-positive samples (optimally, patient samples with known variants should be used). This value is referred as to limit of detection (LoD) and is set based on the required confidence with respect to false-positive and false-negative result probabilities. The greater the distance between LoD and LoB is, the higher the confidence is that the variant is true; on the other hand, the probability of false-negative result increases. Either the overall LoD of the whole assay is estimated (e.g. ensuring truly calling of 99% of all variants), or a variant-specific LoD is set (an approach used by most research studies [4–6, 9]). Assessing LoD and LoB is particularly challenging in the case of *TP53* assessment as the variants can occur in nearly any nucleotide position of *TP53* gene and it is virtually impossible to test all of the potentially existing pathogenic variants at various VAFs; this is even more complicated for variants other than SNVs – e.g., short insertions/deletions. Therefore, the LoD represents only an estimation, and the higher the number of tested variants is, the more precise the estimation is. The set of tested variants should include not only missense variants, but also deletions and insertions, ideally in different gene positions.

Other parameters to be described involve repeatability, reproducibility, and wide range of predictive values. For details, see Supplementary Table S3 and refer to special literature [69, 70, 80, 81].

#### Continuous monitoring of quality

**Fig. 4:**
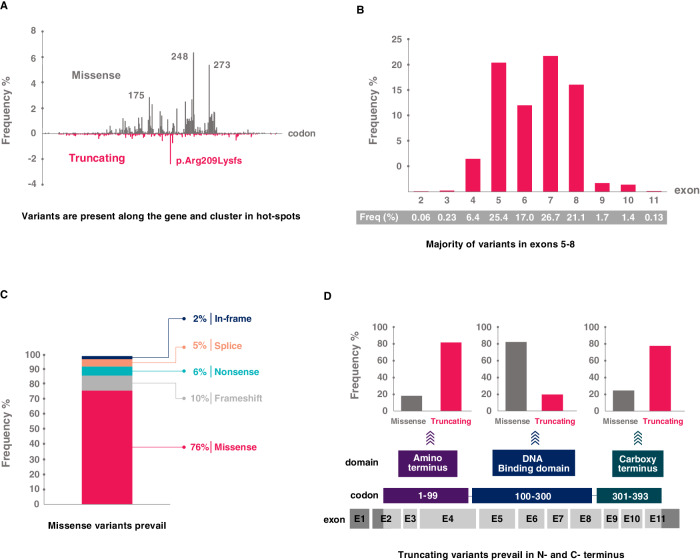
Spectrum of *TP53* defects detected in CLL. *TP53* variant profile based on data collected for CLL patients in the UMD database; common polymorphisms have been omitted [82]. **A** Codon distribution with hot-spot variants depicted. Variants in codons 175, 248, and 273 are general hot spots, while the truncating frameshift variant in codon 209 is CLL-specific. **B** Exon distribution showing the prevalence of variants in exons 5–8. **C** Proportion of variant types out of all variants. **D** Proportion of variant types in individual domains. In the DNA-binding domain, missense variants prevail; conversely, truncating variants are predominant in the carboxy and amino termini.

The performance of the method should be continuously monitored in clinical routine diagnostics. The error rate of each run and sample should be checked. It is recommended to run the same samples repeatedly over an extended period [69] and to perform periodic analyses of reference samples. It is advisable to record all the obtained results in an internal database. It enables following the presence of variants in consecutive samples of individual patients and monitoring the concordance of the obtained results with published data and databases. Repeatedly observed atypical results might suggest an erroneous workflow. Specifically, attention should be paid to the frequency and mutual association of *TP53* mutations and 17p deletions, frequency of low-burden mutations, and *TP53* mutation profile, which is similar to other cancers with a very few exceptions, such as a high prevalence of variant c.626_627del p.Arg209Lysfs in CLL [82] (Fig. 4).

Regular participation in external quality assessment should be standard and is required by ISO 15189. For instance, ERIC cooperates with GenQA/UK NEQAS-LI to assure the quality of *TP53* testing in patients with CLL: ERIC *TP53* Certification ensures the initial control of the method implementation, including the detection of low-VAF *TP53* variants (http://www.ericll.org/), while GenQA/UK NEQAS-LI supports the continuous quality check. Furthermore, ERIC has assisted with interlaboratory comparison of low-VAF variants [66] and will further support such activities.

## Interpretation of the results and reporting

### Variant description

Detected variants must be described using the nomenclature devised by the Human Genome Variation Society (HGVS) [83]. Software tools are helpful to ensure adherence to standardized nomenclature: Mutalyzer [84], or *TP53*-specific tool Seshat, with Mutalyzer embedded [85].

Attention must be paid to the mRNA transcript provided by the bioinformatics pipeline. The preferred reference sequence is the transcript suggested by the MANE project (Ensembl or NCBI) [86] as new Locus Reference Genomic sequences (LRGs) are no longer generated.

Terminology note – the term “variant” is the only acceptable designation in the germline context. For somatic variants, the term “mutation” can be used [83]. From the molecular point of view, somatically gained variants are true “mutations.” Even though the somatic origin is not proven in tumor-only mode, the vast majority of the *TP53* variants found in patients with CLL are truly somatic. Therefore, using the term “mutation” is acceptable for the sake of simplification in clinical utilization in CLL.

### Variant interpretation

Variant interpretation is an integral part of cancer diagnostics. Several consortia have published guidelines for the classification of variants addressing their functional impact and clinical implications (Supplementary Table S4). For germline variants, A joint consensus recommendation of the American College of Medical Genetics and Genomics and the Association for Molecular Pathology (ACMG/AMP) [87] became a standard for classification into five pathogenicity classes. Expert panels for specific genes/diseases further refine these guidelines by providing recommendations for particular genes/diseases (e.g., ClinGen Expert Panel for *TP53* [88]). For somatic variants, distinct classification systems have been published with the aim of defining pathogenicity [89], oncogenicity [90], clinical significance [91] or clinical actionability [92], and modified versions have been issued by national societies [93]. As a result, this situation might cause confusion, and no standardization regarding variant classification and terminology currently exists. Regardless of the classification system applied, it is necessary to adhere to the terminology of the classification system mentioned in the report.

To assist variant interpretation, a plethora of ever-evolving databases, in silico predictors, and aggregation tools are available, many of them designed to be embedded in the bioinformatics pipelines for NGS data analysis (reviewed in [94]). Data obtained through the use of these general tools can assist with the classification of variants detected in larger sets of genes but are often insufficient, or even incorrect. Especially, *in-silico* tools do not work well in the case of *TP53* variants. Moreover, submissions may not be subject to a level of curation sufficient for clinical diagnostic application e.g., different pathogenic *TP53* variants are falsely included in dbSNP databases.

For the purposes of *TP53* analysis in CLL, ERIC standards require using *TP53*-specific databases (see details below) with the support of tools listed in Supplementary Table S5. Overall, we believe that the interpretation workflow might be significantly simplified for the following reasons: (i) *TP53* is the most studied tumor suppressor gene and detailed functional data on transactivation ability [95], loss of growth suppression [96, 97], and dominant negative effect [96] are available for virtually all missense *TP53* variants. These data from large-scale studies are easily accessible via *TP53*-specific databases: the *TP53* database (https://TP53.isb-cgc.org/ originally IARC database) [98], and, the *TP53* website (https://p53.fr/) [99] with the tool Seshat [85]; (ii) from the point of clinical significance and actionability, all somatic *TP53* variants impairing function, i.e. (likely) pathogenic/oncogenic variants, found in patients with CLL are assigned to Tier I **-** *Variants of Strong Clinical Significance* [91], and *Target suitable for routine use* [92]; (iii) the vast majority of *TP53* variants detected in CLL are pathogenic or likely pathogenic [82] and the difference between these two categories does not impact on clinical decision-making in patients with CLL; (iv) when deciding about the oncogenicity/pathogenicity of difficult-to-interpret variants, evidence from hereditary cancer syndromes might be applied [90]. Any germline variant proven to be pathogenic or benign according to the “germline” criteria can be interpreted accordingly when seen as somatic. In this respect, ClinGen *TP53* Variant Curation Expert Panel specifications [88] and the ClinGen Evidence Repository of curated variants (https://erepo.clinicalgenome.org/evrepo/ui/classifications?matchMode=exact&gene=TP53) are assistive.

**Fig. 5:**
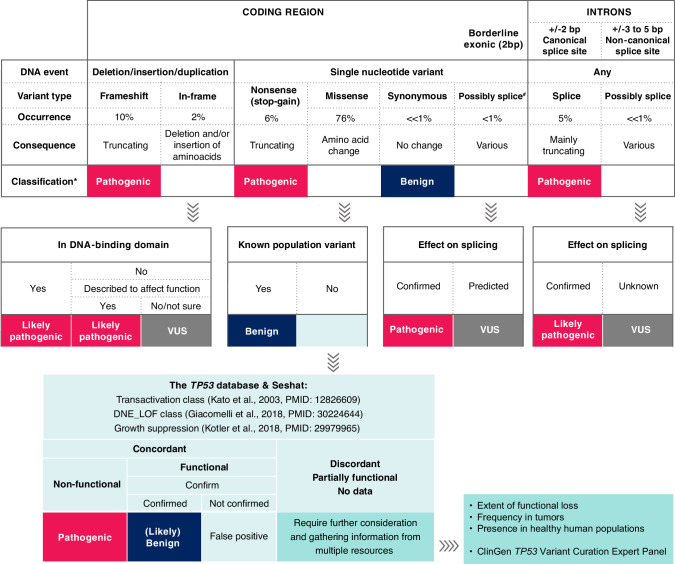
Classification of *TP53* variants detected in CLL. A classification algorithm showing the basic principles of assigning variants into pathogenicity/oncogenicity classes. A detailed version of the algorithm listing assistive tools and specific variants classified into respective categories can be found in Supplementary Figure 1. Databases instrumental in the interpretation of *TP53* variants are listed in Supplementary Table S5. # Might be misclassified as synonymous or missense and listed as such in some databases. *Oncogenicity classification according to Horak et al. [90] is also acceptable. Occurrence according to the UMD database [82]. VUS variant of unknown significance.

**Fig. 6:**
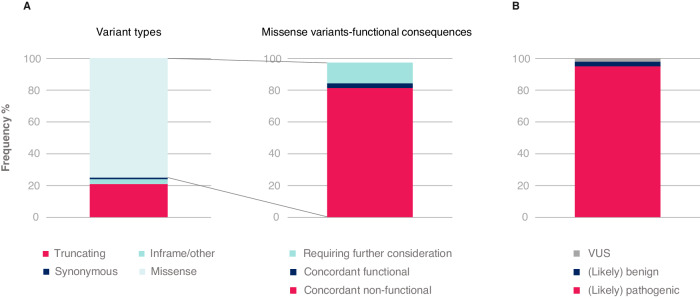
*TP53* variants detected in CLL with respect to their type and interpretation. Illustrative example based on data published in Malcikova et al. [6]. Common population variants have been excluded. **A** Breakdown based on assignment using proposed classification algorithm (color coding corresponds to Fig. 5). Concordant functional/non-functional: assessed by functional tests (Kato et al. [95], Giacomelli et al. [96] and Kotler et al. [97]). **B** Proportion of *TP53* variants detected in CLL assigned to pathogenicity categories. VUS variant of unknown significance.

On these grounds, ERIC proposes for CLL a simplified classification algorithm in which null variants and variants with concordant results from functional studies [95–97] could be classified right away as pathogenic/oncogenic without complicated and time-consuming specification of the criteria (Fig. 5 with more details in Supplementary Figure 1 and notes and clarifications in Supplementary Table S6). This covers most somatic *TP53* variants found in CLL in routine practice. A more detailed evaluation of the oncogenicity/pathogenicity is required only for a minority of the variants (Fig. 6A). Variants with preserved functionality, i.e., (likely) benign variants are infrequent in the somatic context in CLL (Fig. 6B), and such finding is indicative of either germline origin or technical artifact. However, we cannot entirely exclude the presence of a passenger functional *TP53* variant or rare cases of variants of unknown significance. We must admit that p53 functions in the cell are highly complex, therefore, the effects of individual missense mutations are context-dependent [97]. Nevertheless, we believe that a certain degree of simplification is necessary for the purposes of routine CLL diagnostics.

#### Non-tumor DNA testing

CLL is a late-onset cancer not belonging to the Li-Fraumeni syndrome tumor spectrum, and the probability that the detected pathogenic variant in the *TP53* gene is of germline origin is extremely low. Thus, a test to confirm/exclude somatic origin is not generally recommended [100], even for variants with VAF ≥ 50%, as this is a common finding in CLL. In very rare cases, germline origin of (likely) pathogenic variants might be suspected based on clinical information (e.g., presence of family/personal history of Li-Fraumeni-associated cancer and/or exceptionally young age of CLL onset - <40 years); in this case, testing of non-tumor DNA might be considered. In case of suspicion, the patient should be referred to a clinical geneticist before reaching any conclusion on hereditary cancer syndrome testing [101]. Confirming the germline origin must conclude a thorough review of pathogenicity, as a pathogenic variant has far-reaching consequences for the patient and their family.

If indicated, testing of germline origin in patients with CLL should be performed from a non-tumor sample. Given the challenge of obtaining cultured skin fibroblast - the gold standard for germline testing in hereditary hematopoietic malignancies [102] - using an alternative material is acceptable. This can be one of: sorted T cells/CD19-negative fraction (absence of leukemic cells confirmed by flow cytometry), remission samples, buccal swabs/saliva, or other tissues according to the local policy. However, it is essential to keep in mind that also putative tumor-free material (i.e. saliva or CD19 negative blood cells) can be contaminated by CLL cells [103], active myeloid malignancy precursors (e.g., therapy-related myelodysplastic syndrome [104] or myeloproliferative neoplasm) or clonal hematopoiesis of indeterminate potential. Allelic frequency of >30% (SNVs) or >20% (small insertions/deletions) in non-tumor tissue is expected for variants of germline origin [100], and lower VAFs are indicative of cancer cell contamination or, rarely, mosaicism. When the germline origin of the pathogenic *TP53* variant is suspected based on non-tumor sequencing, it is advisable to confirm the result from independent tissue, according to the guidelines for testing in hematopoietic malignancies [102].

### Reporting

The report should be concise and straightforward, while at the same time including all available information that could be relevant to the referring clinician. The obligatory information is summarized in an update of the European Society of Human Genetics (ESHG) recommendations for reporting the results of diagnostic genetic testing [105]. Reports should adhere to the international standard ISO 15189 [68] with the specifications formulated by national accreditation bodies. The template form is provided as Supplementary material but check for the most updated version on www.ericll.org.

Important points to consider when creating a report include the following:

- The cell separation method must be specified in the report. If CD19^+^ cell separation has not been performed, we recommend to include a statement that the result should be interpreted with respect to the proportion of tumor cells in the sample and the separation method used, as a low proportion of tumor cells may lead to a false-negative result or a decreased VAF.
- A clear and brief description of the method and its limitations should be provided, e.g., most sequencing methods are not designed to detect long insertions and deletions spanning whole exons or introns.
- The lowest VAFs that can be reliably detected should be indicated to inform the clinician at which cut-off level the majority of variants is called. This information is essential particularly when issuing negative results.
- Coverage of the whole coding region must be reported (≥99% minimum coverage). Since the *TP53* gene is short and easily covered, covering all bases in the coding region with a sufficient number of reads should be a standard.
- Estimating allele status based on VAF should be avoided (50% VAF can be heterozygous, hemizygous, or homozygous depending on cancer cell fraction and separation method). Also, the VAF does not equal the number of affected cells.
- A brief conclusion summarizing the possible prognostic impact or resistance is recommended to be included in the report along with a reference to the corresponding literature. The content of this conclusion should follow national policies as differences exist between countries regarding the responsibility of the laboratory and the clinician.
- Due to the very low probability of finding a (likely) pathogenic *TP53* variant of germline origin, it is discouraged to suggest in the report the possibility of Li-Fraumeni or other cancer hereditary syndrome (see section “Interpretation, Non-tumor DNA testing”). We recommend mentioning the fact that *“the method cannot distinguish between somatic and germline variants”* among method limitations.

## Summary

Chemoimmunotherapy (CIT) is no longer an option for patients with a *TP53* aberration, irrespective of the clone size. Treatment with targeted agents might prevent the undesirable expansion of *TP53*-mutated clones accompanied by the evolution of other aberrations (e.g. complex karyotype). Nevertheless, data on *TP53* mutations is still evolving in the targeted agent setting and the evidence is not yet mature enough to guide treatment choices among targeted agents (e.g. BTKi and BCL2i) or regimens. ERIC emphasizes the importance of precise classification of *TP53* aberrations (del(17p) vs. *TP53* mutation, mono- vs. biallelic aberrations), as well as inclusion low-VAF *TP53* variants in the design of clinical trials in order to obtain robust evidence for improving the treatment tailoring.

We recommend reporting all *TP53* variants above the LoD set by the laboratory. We emphasize the need for method validation or verification to provide a reliable result, especially in the case of low-VAF variants. It is important for the diagnostic laboratories to adhere to ISO standards. Regarding variant interpretation, most *TP53* variants detected in CLL are unambiguously pathogenic but, in a few instances, the interpretation is less straightforward. We summarized the available information into an algorithm in which the majority of *TP53* variants are classified directly, and we here provide a guide for the interpretation of the less common ambiguous variants. ERIC will continue educational and harmonizing efforts to facilitate robust *TP53* assessment in CLL by organizing educational seminars and QC initiatives and operating an ERIC *TP53* helpdesk for laboratories seeking assistance available at www.ericll.org.

### Supplementary information

Supplementary material

Supplementary Table S2
