# Supplementary material for: ERIC recommendations for TP53 mutation analysis in chronic lymphocytic leukemia—2024 update
Source: Leukemia. 2024 May 16;38(7):1455–68. doi: 10.1038/s41375-024-02267-x (PMC11217004; doi:10.1038/s41375-024-02267-x)
Supplement: Supplementary file 1 — Supplementary material [file 41375_2024_2267_MOESM1_ESM.pdf]

ERIC recommendations for *TP53* mutation analysis in chronic lymphocytic leukemia — 2024 update

Supplementary data

Supplementary Tables

Supplementary Table S1. Summary of studies analyzing the clinical impact of low-VAF *TP53*-mutated clones. 2

Supplementary Table S2. List of primers with highlighted population variants (provided as separate excel file)

Supplementary Table S3. Parameters describing the performance of the NGS test assessed within validation. 4

Supplementary Table S4. List of published guidelines and recommendations for variant description, interpretation and reporting. 5

Supplementary Table S5. List of databases instrumental in the interpretation of somatic *TP53* variants. 6

Supplementary Table S6. Details specifying the classification of *TP53* variants detected in CLL patients 7

Supplementary Figures

Supplementary Figure 1. Detailed classification algorithm of *TP53* variants detected in CLL................9

Other supplementary material

Template report form 10

Supplementary references 12

Supplementary Table S1. Summary of studies analyzing the clinical impact of low-VAF *TP53*-mutated clones.

Parameters of the study									Overall survival (median months/5 year OS*)					Progression-free survival (median months)				
Disease stage	Reference	NGS LOD (VAF)	Cohort type	% U-CLL	Number of patients				*TP53 wt*	*TP53* mut <10% VAF	*TP53* mut ≥10% VAF	Pairwise comparison		*TP53 wt*	*TP53* mut <10 % VAF	*TP53* mut ≥10 % VAF	Pairwise comparison	
					Total	*TP53 wt*	*TP53* mut <10 % VAF	*TP53* mut ≥10 % VAF				*TP53* mut <10% VAF vs wt	*TP53* mut <10 % vs ≥10 % VAF				*TP53* mut <10% VAF vs wt	*TP53* mut <10 % vs ≥10 % VAF
Early stage	Rossi et al. 2014[1]	0.3 %	At diagnosis	36	309	263	18	28	75 %*	46%	35 %*	**p=0.0042**	p=0.6926					
	Nadeu et al. 2016[2]	0.3 %	Untreated (50% at dg)	43	405	361	16	28	82 %*	64%*	54 %*	**p=0.0110**	p=0.4400					
	Brieghel et al. 2019[3]	0.2 %	At diagnosis	32	290	245	25	20	NR	NR	60	p=0.9300	Not shown					
	Bomben et al. 2021[4]	1%	At diagnosis	42	539	467	31	41	NR	93	61	**p=0.0005**	p=0.1241					
At the time of treatment	Rossi et al. 2014[1]	0.3 %	Retrospective	68	53	36	6	11	54 %*	0 %*	12 %*	**p=0.0051**	p=0.4170					
	Brieghel et al. 2019[3]	0.2 %	Retrospective	70	61	44	10	7	72	14	26	**p=0.0020**	Not shown					
	Blakemore et al. 2020[5]	2%	UK LRF CLL4	63	499	440	16	43	73	51	26	p=0.1200	p=0.3290	26	23	6	p=0.1960	p=0.3040
	Malcikova et al 2021[6]	0.1 %	Retrospective	72	511	370	82	59	68	41	22	**p=0.0004**	**p<0.0001**	25	20	7	p=0.0830	**p<0.0001**
	Bomben et al. 2021[4]	1%	Retrospective	55	552	449	42	61	NR	62	47	**p<0.0001**	p=0.3170					
			ARCTIC/ADMIRE	57	251	211	18	22	108	76	31	**p=0.0058**	p=0.3337	69	40	24	**p=0.0045**	p=0.1504

LOD - limit of detection

U-CLL - Unmutated IGHV genes

Supplementary Table S2. List of primers with highlighted population variants (provided as separate excel file).

Supplementary Table S3. Parameters describing the performance of the NGS test assessed within validation. The terminology is inconsistent throughout the literature[7]; it is advisable to explain the calculation briefly alongside the reported parameter. Here, we adopted definitions and procedure from Clinical and Laboratory Standards Institute (CLSI)[8] and A Joint Consensus Recommendation of the Association for Molecular Pathology and College of American Pathologists[9].

**Limit of Blank** (LoB)<br>Definition: Highest measurement result that is likely to be observed for a blank sample. Establishing LoB enables distinguishing of true variants form background noise.<br>Procedure: Per position background distribution estimated based on repeated library preparation and sequencing of negative controls (background VAF does not follow normal distribution). LoB should be set to a value where the false positive rate is close to zero.<br>Ideally: testing of 30 variant-negative samples in duplicate using two reagents lots in independent runs = 120 measurements[8].
**Limit of Detection** (LoD)<br>Definition: The minimum allele fraction that can be detected with a required level of confidence.<br>Procedure: Testing of variant-positive samples (optimally, patients' samples with known variants), serially diluted with variant-negative sample.<br>Ideally: at least five-fold dilution near the target LoD, in 10 replicates analyzed using two reagents lots in two independent runs = 100 measurements); include SNV, small insertions and deletions[8]. Different variants might be pooled in one sample.<br>LoD is set to a safe distance from LoB considering distribution of measured values (low-level somatic VAF do not follow a normal distribution), and the required probability of FP and FN errors<br>Either the overall LoD of the whole assay or variant-specific LoD is estimated.
**Precision**<br>Definition: Closeness of agreement between independent test results.<br>**Replicability/repeatability** = within-run precision<br>Definition: How the test result varies under the same operating conditions.<br>Procedure: Testing the same samples repeatedly within the same run.<br>**Reproducibility** = between-run precision<br>Definition: How the test results vary under different operating conditions.<br>Procedure: Testing the same samples repeatedly in different runs under different conditions (personnel/equipment/reagent lots, etc.)
**Additional parameters**<br>Definition: The wide list of parameters that can be defined includes: **sensitivity, specificity, positive/negative predictive value, false positive/negative rate, accuracy**.<br>Procedure: Running reference samples with known variants, assessing the rate of TP, FP, FN, TN, and calculating the respective values. To obtain robust estimation, as many variables as possible should be included. The most important parameters to be described:<br>**Sensitivity** (alternatively Analytical sensitivity, or Positive Percentage Agreement [PPA], or Recall) = TP/(TP+FN). The proportion of known variants that are detected. The value reaches 1 if all variants present in the reference sample are detected<br>**Positive Predictive Value (PPV)** = TP/(TP+FP). The proportion of detected variants that are true. The value reaches 1 if no false positive results are reported.<br>Note: Assessing PPV is preferred to express the probability of FP call. Calculating specificity using the traditional formula (TN/TN+FP) may lead to a high value masking FP calls due to defining TN as any reference base called as wt.<br>**Caution:** The obtained values depend on the composition of the reference sample; if all tested variants are of high VAF (e.g. >50%), the estimated values will not reflect the method's performance with respect to variants closer to LOD.

FP - false positive, TP - true positive, FN - false negative, TN - true negative

Supplementary Table S4. List of published guidelines and recommendations for variant description, interpretation and reporting.

Recommendation topic and aim		Specifications and notes	Context
**Variant description**			
den Dunnen et al., Hum Mutat 2016[10]	HGVS Recommendations for the Description of Sequence Variants: 2016 Update		
**Interpretation**			
Richards, S et al., Genet Med 2015[11]	Standards and guidelines for the interpretation of sequence variants: A joint consensus recommendation of the American College of Medical Genetics and Genomics and the Association for Molecular Pathology.	5 Class system:<br>Pathogenic<br>Likely Pathogenic<br>Uncertain significance<br>Likely benign<br>Benign	germline
Fortuno et al., Hum Mutat 2021[12]	Specifications of the ACMG/AMP variant interpretation guidelines for germline TP53 variantsɫ	*TP53* specific nuances to classify *TP53* variants into 5-class system formulated by ClinGen TP53 Variant Curation Expert Panel	germline, *TP53* specific
Li et al., JMD 2017[13]	Standards and guidelines for the interpretation and reporting of sequence variants in cancer: a joint consensus recommendation of the Association for Molecular Pathology, American Society of Clinical Oncology, and College of American Pathologists.	4 Tier system:<br>I: Variants of Strong Clinical Significance<br>II: Variants of Potential Clinical Significance<br>III: Variants of Unknown Clinical Significance<br>IV: Benign or Likely Benign Variants	somatic
Horak et al., Genet Med 2022[14]	Standards for the classification of pathogenicity of somatic variants in cancer (oncogenicity): Joint recommendations of Clinical Genome Resource (ClinGen), Cancer Genomics Consortium (CGC), and Variant Interpretation for Cancer Consortium (VICC)	5 categories:<br>Oncogenic<br>Likely oncogenic<br>Variant of uncertain significance<br>Likely benign<br>Benign	somatic
**Reporting**			
Deans et.al., EJHG 2022[15]	Recommendations for reporting results of diagnostic genomic testing		

ɫ For regular updates, please check the TP53-expert panel website: https://clinicalgenome.org/affiliation/50013/

Supplementary Table S5. List of databases instrumental in the interpretation of somatic *TP53* variants.

Database name	Website	Content	Variant origin	Details
The TP53 database[16]	https://tp53.isb-cgc.org/	*TP53* specific	Somatic and germline	Compiles data from the literature and databases on human *TP53* gene variations related to cancer.
The TP53 website (UMD TP53 database)[17, 18]	https://p53.fr/	*TP53* specific	Somatic and germline	Compiles data from the literature and databases on human *TP53* gene variations related to cancer.
Seshat[19]	http://vps338341.ovh.net/			Seshat, tool for variant classification embedded in the TP53 website
Clingen TP53 expert panel[12]	https://erepo.clinicalgenome.org/evrepo/ui/classifications?matchMode=exact&gene=TP53	*TP53* specific	Germline	Contains expert-curated assertions regarding variants' pathogenicity and supporting evidence summaries.
ClinVar[20]	https://www.ncbi.nlm.nih.gov/clinvar/	General	Germline	Publicly available database of gene variant classifications
Cancer hotspots[21]	http://cancerhotspots.org	General	Somatic	Recurrently mutated positions identified in > 24,000 tumor samples
gnomAD (non-cancer)[22]	https://gnomad.broadinstitute.org/	General	Germline	Variant found in individuals with no known personal history of the disease
FLOSSIES	https://whi.color.com/	27 genes known or suggested to harbor mutations that predispose to breast cancer.	Germline	Variants found in women without a personal history of cancer by age ≥70

Supplementary Table S6. Details specifying the classification of *TP53* variants detected in CLL patients.

**Truncating = null variants**	
Specification	Frameshift insertions/deletions, nonsense variants
Rationale	Termination of translation due to the formation of a premature stop codon leads to non-functional protein. Transcripts bearing premature stop codons are often degraded via nonsense-mediated RNA decay resulting in decreased protein level
Interpretation	**Always pathogenic (oncogenic)** irrespective of their presence/absence in databases.
**Splice site variants - canonical**	
Specification	Variants in +/-2 intronic bases
Rationale	Most splice site variants in the *TP53* gene lead to frameshift (see null variants). Only variants in the acceptor site of intron 3 (preceding exon 4) theoretically lead to in-frame exon skipping, but various aberrantly spliced transcripts might be formed.
Interpretation	**Always pathogenic (oncogenic)**
**Splice site variants – noncanonical**	
Specification	Variants in +/-5 intronic bases
Rationale	Variants in nucleotides adjacent to canonical splice sites might affect splicing. The evidence can be found at the MutSpliceDB (https://brb.nci.nih.gov/splicing). For the *TP53* gene, so far, only variants in position c.375+5 have documented an impact on splicing[23], but a continuous extension of the data may be expected.
Interpretation	Variants with **experimental evidence documenting splicing defect** - **likely pathogenic (oncogenic).**
**Intronic and UTR variants**	
Specification	Variants in inner parts of introns further from splice sites and in 3' and 5'UTR regions
Rationale	Variants may affect transcription or splicing, but there is generally lack of evidence and large number of population variants are located in these regions.
Interpretation	**Analysis is not recommended** in routine practice.<br>Might be classified as likely pathogenic (oncogenic) if experimental evidence exists
**In-frame variants**	
Specification	Deletions and insertions not disturbing the reading frame
Rationale	Limited data on the functional impact exist. The vast majority of so-far analyzed in-frame deletions within the DNA-binding domain showed impaired anti-proliferative capacity in H1299 cells[24]. The functional data are to be found via The *TP53* database for some but not all tested variants. ERIC initiated the study of the impact of the in-frame variants found in patients with CLL; all 45 tested variants showed disturbed functionality (manuscript in preparation).
Interpretation	**Within the DNA-binding domain - likely pathogenic (oncogenic)**<br>**Outside the DNA-binding domain - VUS** unless functional data or proven association with Li-Fraumeni or hereditary cancer syndromes exist.
**Synonymous variants**	
Specification	Single-nucleotide change not leading to amino acid change
Rationale	Not all synonymous variants are silent. The variants present at the exon-intron boundary might affect splicing[25] (see Supplementary Figure 1). Care must be taken not to exclude all synonymous variants during the filtering in bioinformatics pipelines.
Interpretation	Most are **benign** but specific variants affecting splicing are **pathogenic (oncogenic).**

**Missense variants**	
Specification	Single-nucleotide change variants leading to amino acid change
Rationale	Amino-acid change mostly affects p53 protein structure or DNA binding ability.
Interpretation	Variants with concordant data from functional studies[24, 26, 27] can be directly interpreted as **(likely) pathogenic (oncogenic) -** most variants, or **(likely) benign** - minority of variants. For variants with discordant or lacking functional data further consideration is required (see Supplementary Figure 1).<br>Caution: A few specific variants located in borderline exon/intron nucleotides affect splicing although they were classified as functional (see Supplementary Figure 1).<br>**Population variants:** Several missense variants occur in a human population without affecting p53 function. Variant c.215C>G p.Pro72Arg is the most common benign SNP, others are listed in Supplementary Figure 1. The rare variants should be checked using Clingen repository[28] and GnomAD[22].

Supplementary Figure 1. Detailed classification algorithm of *TP53* variants detected in CLL.
Databases instrumental in the interpretation of *TP53* variants are listed in Supplementary Table S5.
# Might be misclassified as synonymous or missense and listed as such in some databases.
* Oncogenicity classification according to Horak et al. 2022[14] is also acceptable.
Occurrence according UMD database[18].

	CODING REGION						INTRONS	
						**Borderline exonic (2 bp)**	**+/-2 bp Canonical splice site**	**+/-3 to 5 bp Non-canonical splice site**
**DNA event**	**Deletion/insertion/duplication**		**Single nucleotide variant**				**Any**	
**Variant type**	**Frameshift**	**In-frame**	**Nonsense (stop-gain)**	**Missense**	**Synonymous**	**Possibly splice#**	**Splice**	**Possibly splice**
**Occurrence**	10%	2%	6%	76%	<<1%	<1%	5%	<<1%
**Consequence**	Truncating	Deletion and/or insertion of aminoacids	Truncating	Amino acid change	No change	Various	Mainly truncating	Various
**Classification***	Pathogenic		Pathogenic		Benign		Pathogenic	

In DNA-binding domain (In-frame)

Yes	No	
	Described to affect function<br>• Databases<br>• Functional data<br>• Literature<br>• ERIC helpdesk	
	Yes	No/not sure
Likely pathogenic	Likely pathogenic	VUS

Known population variant (Missense)

Yes	No
c.91G>A p.V31I<br>c.108G>A p.P36=<br>c.139C>T p.P47S<br>c.215C>G p.P72R<br>c.639A>G p.R213=<br>c.704A>G p.N235S<br>c.869G>A p.R290H<br>…	
Benign	

Effect on splicing (Possibly splice#)

Confirmed	Predicted
c.375G>A p.T125=<br>c.375G>T p.T125=<br>c.375G>C p.T125=<br>c.559G>C p.G187R<br>c.672G>A p.E224=<br>c.672G>T p.E224D<br>c.782G>C p.S261T	Multiple variants
Pathogenic	

Effect on splicing (Possibly splice, introns)

Confirmed	Unknown
c.375+5G>A p?<br>c.375+5G>C p?<br>c.375+5G>T p?<br>Others:<br>• Databases<br>• Functional data<br>• Literature<br>• ERIC helpdesk	
Likely pathogenic	

The *TP53* database & Seshat:
Transactivation class (Kato et al., 2003, PMID: 12826609))
DNE_LOF class (Giacomelli et al., PMID: 30224644)
Growth suppression (Kotler et al. ,2018, PMID: 29979965)

**Concordant**			**Discordant<br>Partially functional<br>No data**
**Non-functional**	**Functional**		
	Confirm		
	Confirmed	Not confirmed	
Pathogenic	(Likely) Benign	False positive	Require further consideration and gathering information from multiple resources

Consider:
- Extent of functional loss
- Frequency in tumors
- Presence in healthy human populations
  - gnomAD
  - Doffe et al. 2021 (PMID: 33257846)
  - FLOSSIES
- The ClinGen Evidence Repository by *TP53* Variant Curation Expert Panel
- Final comment from Seshat
- ClinVar interpretation and evidence

Use guidelines for classification of somatic variants (Horak et al. 2022; PMID: 36063163) with the application of *TP53* specific nuances as defined in ClinGen *TP53* Expert Panel Specifications (Fortuno et al., 2021; PMID: 33300245)

Template report form. Please check for the most updated version on www.ericll.org

Logo of the Hospital/Laboratory

Mutational analysis of the *TP53* gene

Performed by
Laboratory name:
Laboratory address:
(full contact details including phone number)

Requested by
Hospital:
Referrer:
Address:

Patient name/id: *
Date of birth:
Gender:
Reason for referral:

Date of sample collection:
Date of sample delivery:
Result issued:

Type of material:
Cell separation:
Sample identification number:
Method:

Result: *TP53* MUTATION DETECTED / NOT DETECTED

Mutation No.	Variant<br>Reference sequence: NC_000017.11<br>(NM_000546.6)	Variant allele frequency (VAF)	Mutation type *(optional)*	Pathogenicity
1				
2				

Optional:
Comparison with a previous sample: *We observed an increase/decrease in variant allele frequency compared to previous sample (sampling date xx.xx.xx variant allele frequency xx%).*

Conclusion:
Example: *A pathogenic variant was found within the TP53 gene. TP53 mutations are associated with adverse prognosis and poor response to chemoimmunotherapy in CLL and therefore such treatment should be avoided (PMID: 33091559 or other reference(s) of current national or international guidelines).*

Or: No pathogenic variant within the TP53 gene was detected

The result should be interpreted with respect to the proportion of tumor cells in the primary sample and the separation method used. A low proportion of tumor cells in the sample may lead to a false negative result or a decreased VAF.

Mutational analysis of the *TP53* gene

Patient name/id: *	Date of sample collection:
Date of birth:	Date of sample delivery:
Gender:	Result issued:
Reason for referral:	

Analytical method description (region sequenced, method description including bioinformatics pipeline):
Minimal coverage of the target region:
Detection limit of the method:
Variants are described according the Human Genome Variation Society (HGVS) nomenclature Version xx.xx.

Variant interpretation:
Functional impact and pathogenicity of variants was assessed based on the following tools:

The interpretation refers to the time of issuing of the report and may change in the future due to additional evidence. Validated polymorphisms and benign/likely benign variants are not included in this report and can be provided upon request.

Method limitations:
Example: *The method cannot detect large duplications and deletions, and complex rearrangements within the tested regions of TP53 gene. The procedure cannot distinguish between somatic and germline variants without testing of normal tissue from the same individual. In the case of justified suspicion of the germinal origin of a variant with VAF>50% (young age, family history), the examination needs to be repeated from non-tumor DNA.*

Analysis performed by[ƚ]: Name and function   Result issued by: Name and function[ƚ]

** Unique patient identification, the date of primary sample collection and the date of the issue of the report should be on each page of the report (in a header or a footer of the document).*
ƚ Co-validation and co-signature by a second competent person is recommended (and mandatory in some countries).

Note: Pages should be numbered in a format: 1/2, 2/2

Supplementary references

1. Rossi D, Khiabanian H, Spina V, Ciardullo C, Bruscaggin A, Famà R*, et al.* Clinical impact of small TP53 mutated subclones in chronic lymphocytic leukemia. *Blood* 2014 Apr; **123**(14)**:** 2139-2147.

2. Nadeu F, Delgado J, Royo C, Baumann T, Stankovic T, Pinyol M*, et al.* Clinical impact of clonal and subclonal TP53, SF3B1, BIRC3, NOTCH1, and ATM mutations in chronic lymphocytic leukemia. *Blood* 2016 Apr 28; **127**(17)**:** 2122-2130.

3. Brieghel C, Kinalis S, Yde CW, Schmidt AY, Jønson L, Andersen MA*, et al.* Deep targeted sequencing of T*P53* in chronic lymphocytic leukemia: clinical impact at diagnosis and at time of treatment. *Haematologica* 2019 Apr; **104**(4)**:** 789-796.

4. Bomben R, Rossi FM, Vit F, Bittolo T, D'Agaro T, Zucchetto A*, et al.* Mutations with Low Variant Allele Frequency Predict Short Survival in Chronic Lymphocytic Leukemia. *Clin Cancer Res* 2021 Oct 15; **27**(20)**:** 5566-5575.

5. Blakemore SJ, Clifford R, Parker H, Antoniou P, Stec-Dziedzic E, Larrayoz M*, et al.* Clinical significance of TP53, BIRC3, ATM and MAPK-ERK genes in chronic lymphocytic leukaemia: data from the randomised UK LRF CLL4 trial. *Leukemia* 2020 Jul; **34**(7)**:** 1760-1774.

6. Malcikova J, Pavlova S, Barbara KV, Radova L, Plevova K, Kotaskova J*, et al.* Low-burden TP53 mutations in CLL: clinical impact and clonal evolution within the context of different treatment options. *Blood* 2021 Dec 23; **138**(25)**:** 2670-2685.

7. CLSI Harmonized Terminology Database. [cited; Available from: https://clsi.org/standards-development/harmonized-terminology-database/

8. Clinical and Laboratory Standards Institute (CLSI). Human Genetic and Genomic Testing Using Traditional and High-Throughput Nucleic Acid Sequencing Methods. 3rd ed. CLSI guideline MM09. Clinical and Laboratory Standards Institute, USA; 2023.

9. Jennings LJ, Arcila ME, Corless C, Kamel-Reid S, Lubin IM, Pfeifer J*, et al.* Guidelines for Validation of Next-Generation Sequencing-Based Oncology Panels: A Joint Consensus Recommendation of the Association for Molecular Pathology and College of American Pathologists. *J Mol Diagn* 2017 May; **19**(3)**:** 341-365.

10. den Dunnen JT, Dalgleish R, Maglott DR, Hart RK, Greenblatt MS, McGowan-Jordan J*, et al.* HGVS Recommendations for the Description of Sequence Variants: 2016 Update. *Hum Mutat* 2016 Jun; **37**(6)**:** 564-569.

11. Richards S, Aziz N, Bale S, Bick D, Das S, Gastier-Foster J*, et al.* Standards and guidelines for the interpretation of sequence variants: a joint consensus recommendation of the American College of Medical Genetics and Genomics and the Association for Molecular Pathology. *Genet Med* 2015 May; **17**(5)**:** 405-424.

12. Fortuno C, Lee K, Olivier M, Pesaran T, Mai PL, de Andrade KC*, et al.* Specifications of the ACMG/AMP variant interpretation guidelines for germline TP53 variants. *Hum Mutat* 2021 Mar; **42**(3)**:** 223-236.

13. Li MM, Datto M, Duncavage EJ, Kulkarni S, Lindeman NI, Roy S*, et al.* Standards and Guidelines for the Interpretation and Reporting of Sequence Variants in Cancer: A Joint Consensus Recommendation of the Association for Molecular Pathology, American Society of Clinical Oncology, and College of American Pathologists. *J Mol Diagn* 2017 Jan; **19**(1)**:** 4-23.

14. Horak P, Griffith M, Danos AM, Pitel BA, Madhavan S, Liu X*, et al.* Standards for the classification of pathogenicity of somatic variants in cancer (oncogenicity): Joint recommendations of Clinical Genome Resource (ClinGen), Cancer Genomics Consortium (CGC), and Variant Interpretation for Cancer Consortium (VICC). *Genet Med* 2022 Sep; **24**(9)**:** 1991.

15. Deans ZC, Ahn JW, Carreira IM, Dequeker E, Henderson M, Lovrecic L*, et al.* Recommendations for reporting results of diagnostic genomic testing. *Eur J Hum Genet* 2022 Sep; **30**(9)**:** 1011-1016.

16. de Andrade KC, Lee EE, Tookmanian EM, Kesserwan CA, Manfredi JJ, Hatton JN*, et al.* The TP53 Database: transition from the International Agency for Research on Cancer to the US National Cancer Institute. *Cell Death Differ* 2022 May; **29**(5)**:** 1071-1073.

17. Leroy B, Anderson M, Soussi T. TP53 mutations in human cancer: database reassessment and prospects for the next decade. *Hum Mutat* 2014 Jun; **35**(6)**:** 672-688.

18. Soussi T, Baliakas P. Landscape of TP53 Alterations in Chronic Lymphocytic Leukemia. *Front Oncol* 2022; **12:** 808886.

19. Tikkanen T, Leroy B, Fournier JL, Risques RA, Malcikova J, Soussi T. Seshat: A Web service for accurate annotation, validation, and analysis of TP53 variants generated by conventional and next-generation sequencing. *Hum Mutat* 2018 07; **39**(7)**:** 925-933.

20. Landrum MJ, Lee JM, Benson M, Brown GR, Chao C, Chitipiralla S*, et al.* ClinVar: improving access to variant interpretations and supporting evidence. *Nucleic Acids Res* 2018 Jan 04; **46**(D1)**:** D1062-D1067.

21. Chang MT, Bhattarai TS, Schram AM, Bielski CM, Donoghue MTA, Jonsson P*, et al.* Accelerating Discovery of Functional Mutant Alleles in Cancer. *Cancer Discov* 2018 Feb; **8**(2)**:** 174-183.

22. Karczewski KJ, Francioli LC, Tiao G, Cummings BB, Alföldi J, Wang Q*, et al.* The mutational constraint spectrum quantified from variation in 141,456 humans. *Nature* 2020 May; **581**(7809)**:** 434-443.

23. Chui MH, Yang C, Mehta N, Rai V, Zehir A, Momeni Boroujeni A*, et al.* Somatic intronic TP53 c.375+5G mutations are a recurrent but under-recognized mode of TP53 inactivation. *J Pathol Clin Res* 2022 Jan; **8**(1)**:** 14-18.

24. Kotler E, Shani O, Goldfeld G, Lotan-Pompan M, Tarcic O, Gershoni A*, et al.* A Systematic p53 Mutation Library Links Differential Functional Impact to Cancer Mutation Pattern and Evolutionary Conservation. *Mol Cell* 2018 Sep 06; **71**(5)**:** 873.

25. Supek F, Miñana B, Valcárcel J, Gabaldón T, Lehner B. Synonymous mutations frequently act as driver mutations in human cancers. *Cell* 2014 Mar 13; **156**(6)**:** 1324-1335.

26. Kato S, Han SY, Liu W, Otsuka K, Shibata H, Kanamaru R*, et al.* Understanding the function-structure and function-mutation relationships of p53 tumor suppressor protein by high-resolution missense mutation analysis. *Proc Natl Acad Sci U S A* 2003 Jul 8; **100**(14)**:** 8424-8429.

27. Giacomelli AO, Yang X, Lintner RE, McFarland JM, Duby M, Kim J*, et al.* Mutational processes shape the landscape of TP53 mutations in human cancer. *Nat Genet* 2018 Oct; **50**(10)**:** 1381-1387.

28. https://erepo.clinicalgenome.org/evrepo/ui/classifications?matchMode=exact&gene=TP53.
